# Management of oropharyngeal cancer: a cross-sectional review of institutional practice at a large Canadian referral centre

**DOI:** 10.1186/1916-0216-43-19

**Published:** 2014-06-24

**Authors:** Lindsay Wilson, Danny Enepekides, Kevin Higgins

**Affiliations:** 1 Department of Otolaryngology – Head & Neck Surgery, Research Coordinator, Sunnybrook Health Sciences Centre, 2075 Bayview Avenue, Room M1 123, Toronto, ON, Canada; 2 Department of Otolaryngology – Head and Neck Surgery, Faculty of Medicine, University of Toronto. Microvascular Reconstructive Surgery, Head & Neck Surgical Oncology / Endocrine Surgery. Sunnybrook Health Sciences Centre, 2075 Bayview Avenue, Room M1 102, Toronto, ON, Canada

**Keywords:** Oropharyngeal cancer, Concomitant chemoradiation, Salvage surgery, Free-flap reconstruction, Functional rehabilitation

## Abstract

**Background:**

Over the years, the treatment of oropharyngeal cancer has changed; in the past, first-line treatment consisted of surgery followed by adjuvant radiotherapy, today however, primary treatment typically involves concomitant chemoradiation, and reserves surgery for salvage. While chemoradiation is the modality of choice for primary management of oropharyngeal cancer, disease characteristics, institutional bias, and patient preferences influence treatment choice. This has lead to variation in the treatment of OPC, and has generated some uncertainly regarding the ideal therapeutic approach. The objective of this study was to describe the treatment of OPC a large Canadian referral center, highlighting trends in treatment choice and outcome.

**Methods:**

This is a cross-sectional retrospective review of clinical practice at Sunnybrook Health Science Centre (Toronto, ON). This investigation documents type of first-line treatment, rates of treatment failure, rates of surgical salvage, and 5-year disease-free survival. This study also asses the therapeutic impact of free-flap reconstruction on the use of a postoperative tracheostomy and/or percutaneous endoscopic gastrostomy tube.

**Results:**

The majority of oropharyngeal cancer patients presented with regionally metastatic disease (stage III-IV) and underwent concomitant chemoradiation as first-line treatment. Just over half of patients who failed chemoradiation were eligible for salvage surgery. Forty-six percent of salvage patients recurred at approximately 6 months, and died approximately 12 months following the first sign of disease recurrence. Five-year survival for salvage patients stage II, III, IVA, and IVB was 100%, 54.5%, 53.8%, and 50%, respectively. The incidence of percutaneous endoscopic gastrostomy tubes and tracheostomies was comparable between patients who underwent free-flap reconstruction and patients who did not.

**Conclusion:**

The modality of choice for first-line treatment of oropharyngeal cancer is concomitant chemoradiation. The moderate failure rate following chemoradiation and the modest survival rate following salvage surgery could indicate that selected patients may benefit from undergoing surgery as first-line treatment. While this study did not show that functional outcomes were better for free-flap patients, it is highly likely that those who received a free-flap did better then they would have had they not undergone reconstructive surgery. More research regarding the therapeutic effects of free-flaps in OPC survivors is needed.

## Background

Oropharyngeal cancer (OPC) currently ranks 13^th^ among the most common forms of cancer in Canada, and accounts for 475 newly diagnosed cases of cancer each year. ^a^ Documented risks for OPC include male sex, alcohol and tobacco use, and HPV infection [1-3]. While OPC is more common in individuals 60 years of age or older, the rise in HPV-related cancers has lead to an increase in disease incidence in younger populations [3].

In the past, the treatment of OPC has involved surgery followed by adjuvant radiotherapy (RT). Today, most treatment regimes favor concomitant chemoradiation (CCRT), reserving surgery for salvage. While CCRT is now considered the standard in OPC treatment, disease characteristics, patient preferences, and institutional biases have substantial bearing on treatment decisions. This has lead to institutional differences in first-line treatment of OPC and has generated some uncertainty regarding the ideal therapeutic approach [1].

The principle aim of this study is to describe the treatment of OPC at a tertiary Canadian institution, and add to the Canadian literature on the management and outcomes of OPC. This research describes first-line treatment, the rate of surgical salvage; and outcomes following salvage surgery.

## Methods

### Sample selection

Patients were identified using the Head and Neck Database maintained by the Department of Otolaryngology–Head and Neck Surgery, at Sunnybrook Heath Science Centre (SHSC). The Head and Neck Database contains a subset of patients treated for head and neck cancer at SHSC from January 2004 to January 2013. All patients who presented in the Cancer Center at SHSC with oropharyngeal primaries and squamous cell carcinoma were eligible for inclusion. Patients were excluded if they presented with a second primary that did not involve the oropharynx or if they had initiated cancer treatment prior to their presentation at Sunnybrook. Patient characteristics are provided in the results section of this paper.

### Data collection

All patient data was handled according to standards set forth by the Sunnybrook Research Ethics Board. Electronic patient records, available through the Sunnybrook network, were used to collect patient data. Variables included age at the time of diagnosis, sex, drinking and smoking histories, site of involvement, TNM stage, disease stage, first-line treatment, response to primary treatment, disease outcomes following primary treatment (i.e. disease-free survival and overall survival) time to recurrence following primary treatment, secondary treatment (i.e. salvage surgery), salvage procedures, disease outcomes following salvage surgery (i.e. disease-free survival and overall survival), time to recurrence following salvage surgery, use of a post-salvage percutaneous endoscopic gastrostomy tube (PEG-tube) and/or tracheostomy. All patients were staged using the 6^th^ edition of the American Joint Committee on Cancer (AJCC) 2010 staging system for oropharygeal cancer [4]. Time to recurrence was measured from the completion of primary treatment to the first sign of disease recurrence (clinically or radiologically). Survival was measured from the completion of treatment to the time of death, or 60 months of clinical follow-up. Patients that had clinical reports that ended before the 60-month mark were censored. The presence/absence of PEG-tubes and/or tracheostomies was documented along four different time points: (1) start of treatment, (2) 6 months following the completion of treatment, (3) 12 months following treatment, and (4) 60 months following treatment. Response to treatment was defined as complete, incomplete, or partial. Patients were considered to have a “complete” response if full locoregional control was achieved. Patients who did not respond to treatment, experienced disease progression, or were unable to complete their therapy due to toxicity/intolerable side effects were considered “incomplete”. A patient who had a “partial” response showed signs of improvement following treatment amounting to at least a 50% reduction in tumor volume. Patients who had a complete response and remained disease-free over 60 months of clinical follow-up or more were considered cured. Patients who had a partial response or experienced a recurrence and were not eligible for secondary treatment with curative intent were considered palliative. Patients amenable to further treatment went on to have salvage surgery. All patients were assigned a disease status at the end of their treatment: no evidence of disease (NED), alive with disease (AWD), dead of disease (DOD). If a patient was referred to palliative care, they were considered DOD by the start of the following year.

Due to the fact that HPV testing is currently not yet part of standard practice for the diagnosis and treatment of OPC, very few patient records had this information available. Low reporting of HPV status made an analysis impossible. Therefore this information was not included in this study.

### Analysis

Analyses were performed using IBM SPSS GradPack statistics software, version 21. Results were deemed significant if p < 0.05, and highly significant If p < 0.01. A chi-squared test was used to compare sets of parametric data, and an unpaired students t-test was used to compare the means of independent samples. The survival analysis was conducted using the Kaplan Meier method and differences in survival curves were analyzed using a Mantel-Cox log rank test.

## Results

### Patient characteristics

**Figure 1:**
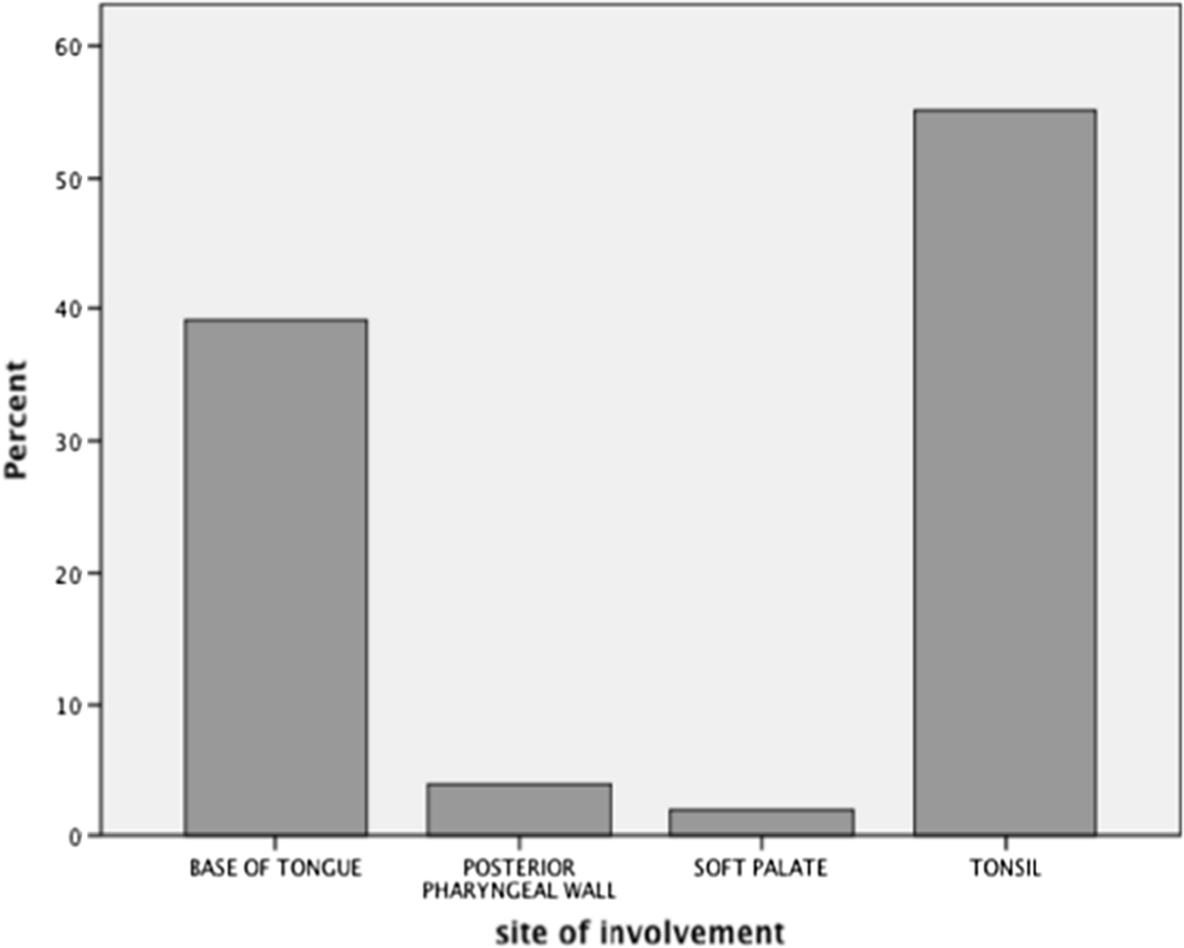
Percentage of patients with OPC who presented with base of tongue, posterior pharyngeal wall, soft palate, and tonsil primaries (n = 153).

One hundred and seventy-seven newly diagnosed oropharyngeal cancer patients were identified and 153 were eligible for inclusion. Patients who were excluded did not have an oropharyngeal primary and/or did not have squamous cell histology, and/or were presenting for the first time to SHSC with recurrent disease. Of all the OPC patients included in this study, 41 (26.9%) were female, and 112 (73.0%) were male. The mean age for all patients was 62.1 (±12.1) years, and ranged from 26 to 93. Twenty-five (16.3%) patients had no history of smoking or drinking, and 129 (83.7%) had a history of smoking and/or drinking; the majority of which (65%) had a history of both smoking and drinking. There were no patients with in situ, stage I, or stage IVC disease. The majority (55.6%) of patients were stage IVA, followed by stage III (27.0%), stage IVB (13.0%), and then stage II (4.6%). ^b^ One hundred and twenty-five patients (81.7%) had regional metastases at first presentation, the majority of which occurred in level II (67.2%). Few patients presented with nodal involvement in more than one neck level (16.8%), and/or extracapsular extension (1.6%). Most OPC patients (55.6%) had tonsil primaries, followed by the base of tongue (38.6%), the posterior pharyngeal wall (3.9%), and then the soft palate (2.0%). The tonsil and base of tongue had a tendency to present with more advanced stage disease compared to the posterior pharyngeal wall and soft palate cancers. Ninety-seven percent of base of tongue cancers were stage III or higher, and 94% of tonsil cancers were stage III or higher. A percent distribution of sub-site is provided in Figure 1. A cross-tabulation of site of involvement and disease stage can be found in Table 1.

**Table 1:** **Cross**-**tabulation of site of involvement and disease stage for oropharyngeal cancer patients treated at Sunnybrook Health Science Centre**

**Site of involvement**	**Disease stage**			
	II	III	IVA	IVB
Base of tongue	2	17	31	9
Posterior pharyngeal wall	0	3	3	0
Soft palate	1	2	0	0
Tonsil	4	19	51	11

Cross-tabulation of site of involvement and disease stage for OPC (n = 153).

### First-line treatment of OPC and the incidence of treatment failure

**Figure 2:**
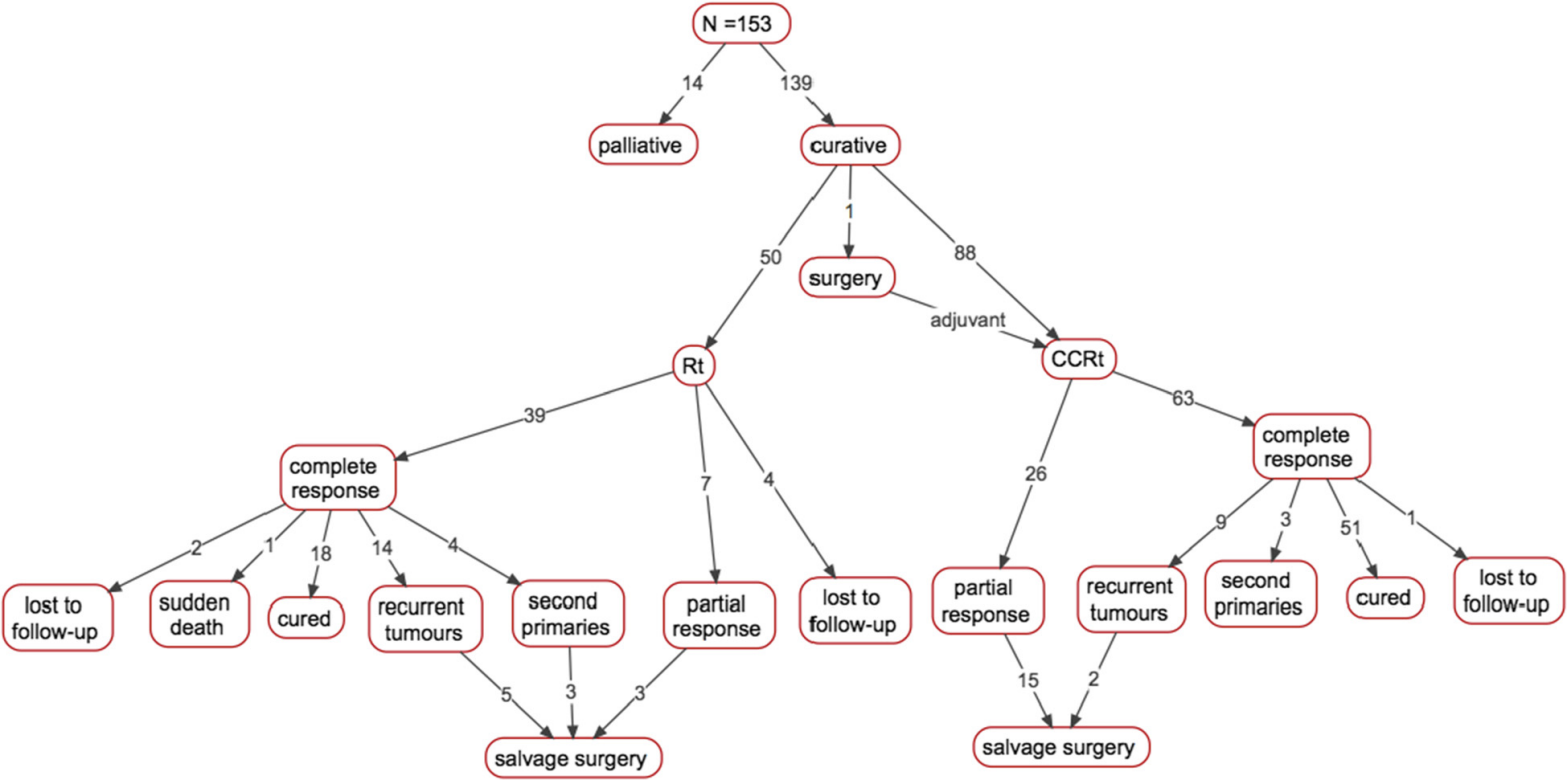
Path model for cohort of OPC patients detailing prognosis at first visit (branch 1), first-line treatment (branch 2), response to treatment (branch 3), and salvage rate (branch 4) (n = 153).

One hundred and thirty-nine patients (90.8% of patients with OPC) were treated with curative intent at first visit, and 14 (9.2%) were considered palliative. Of those who were considered palliative, 10 were stage IVA and 4 were stage IVB. Nearly 80% of patients had T4b tumors, and 85.7% presented with advanced neck disease. The majority of patients also had with significant comorbidity, and/or a low functional status rating. All but one of the patients treated with palliative intent received RT, 3 of these patients died before the completion of their last fraction and the remaining patients died within 24 months of their diagnosis.Fifty patients (36% of patients treated with curative intent) received RT as their primary mode of care. The average dose of RT was 62 Gy (R = 32-70 Gy), delivered in 31 fractions, over 1 to 3 phases. Thirty-nine patients (78% of patients treated with RT) had a complete response, 7 (14%) had a partial response, and 4 were lost to follow-up. Of the patients who had a complete response, 18 (46.2%) were cured of their disease, 14 (35.9%) developed recurrent tumors, and 4 (10.3%) developed second primaries. The average time to recurrence following RT was 13.0 (±11.4) months and ranged from 3 to 38. Eleven patients with treatment failure following RT were considered operable and proceeded with salvage surgery (5 recurrent tumors, 3 second primaries, and 3 residual tumors). Figure 2 provides a complete overview of patient treatment and clinical response.Eighty-eight patients (64% of patients treated with curative intent) received CCRT as their primary mode of care. The average dose of CCRT was 66 Gy (R = 50-70 Gy), delivered in 33 fractions, over 1 to 3 phases. There was only 1 patient that received adjuvant CCRT following surgical resection. Sixty-three patients (71.6% of patients treated with CCRT) had a complete response, and 26 (29.5%) had a partial response. Of those who had a complete response, 51 patients (81%) were cured of their disease, 9 (14.2%) experienced a recurrence, and 3 (4.8%) developed second primaries. The average time to recurrence was 23.7 (±20.1) months and ranged from 3 to 60. Seventeen patients with treatment failure following CCRT were deemed eligible for salvage surgery [2 recurrent tumors, and 15 residual tumors (Figure 2)]. The Sunnybrook experience of surgical salvage will be discussed in the next section.

A chi-squared test was performed to determine whether treatment failure was significantly different for patients who received CCRT as opposed to RT. Results indicate that recurrence rates and the incidence of second primaries were higher for the RT group (35.9% vs. 14.3%; p < 0.001), and the incidence of residual disease was higher for the CCRT group (29.5% vs. 14.0%; p < 0.001). Time to recurrence was comparable between the two groups (p >0.05). A subsequent chi-squared test was performed to determine whether disease characteristics were homogenous between the two treatment groups. Patient characteristics including T-stage and site of involvement were not significantly different for patients who underwent RT as opposed to CCRT. N-stage (p < 0.001) and disease stage (p < 0.001) were significantly different between the two treatment groups. Accordingly, there were more patients with an N-stage <1 and a disease-stage < III in the RT group, and were more patients with an N-stage > 2 and a disease stage > IVA in the CCRT group. A student’s t-test was performed to determine whether the age of patients was significantly different for either of the treatment groups. The mean age of patients undergoing CCRT and RT was 57.7(±10.1) and 67 (±11.3), respectively; this difference was highly statistically significant [t = 9.3 (95%CI: 13.1–5.7), p < 0.001]. Patient survival for the RT and CCRT groups was 65.2% and 54.9%. Differences in survival were not significant.

In summary, patients who received CCRT were generally younger and presented with more advanced disease stage, and specifically more advanced neck disease, compared to patients who received RT alone. See Table 2 for a breakdown of patient characteristic for each of the treatment groups including site of involvement, disease stage, N-stage, and T-stage.

**Table 2:** Patient characteristics for RT and CCRT cohorts

**Site of involvement**	**RT group**	**CCRT group**
Base of tongue	21	33
Posterior pharyngeal wall	2	1
Soft palate	2	1
Tonsil	25	55
**T-stage**	**RT group**	**CCRT group**
1	8	16
2	20	30
3	16	22
4	6	22
N-stage**	**RT group**	**CCRT group**
0	17	9
1	16	20
2	14	48
3	3	13
Disease stage**	**RT group**	**CCRT group**
II	5	2
III	24	17
IVa	4	10
IVA	14	48
IVB	3	13

**Significant to 0.001.

Comparison of site of involvement, T-stage, N-stage, and disease stage of patients who underwent RT (n = 50) as first-line treatment as opposed to CCRT (n = 88); **Significant to 0.01.

### Salvage surgery

**Figure 3:**
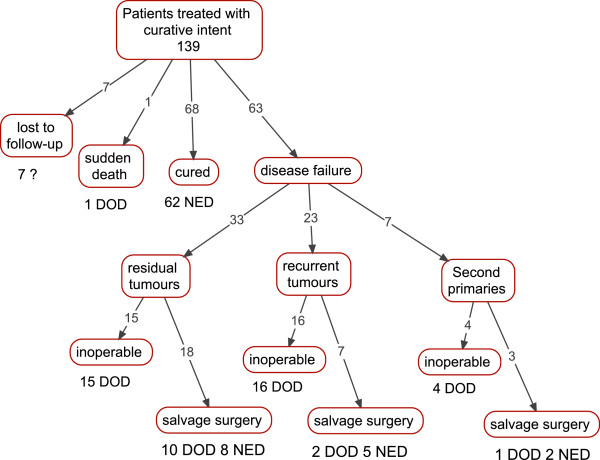
**Summary of clinical response to primary treatment including type of treatment failure, surgical salvage, and disease outcomes following salvage surgery (pooled data from Figure**2**).**

**Figure 4:**
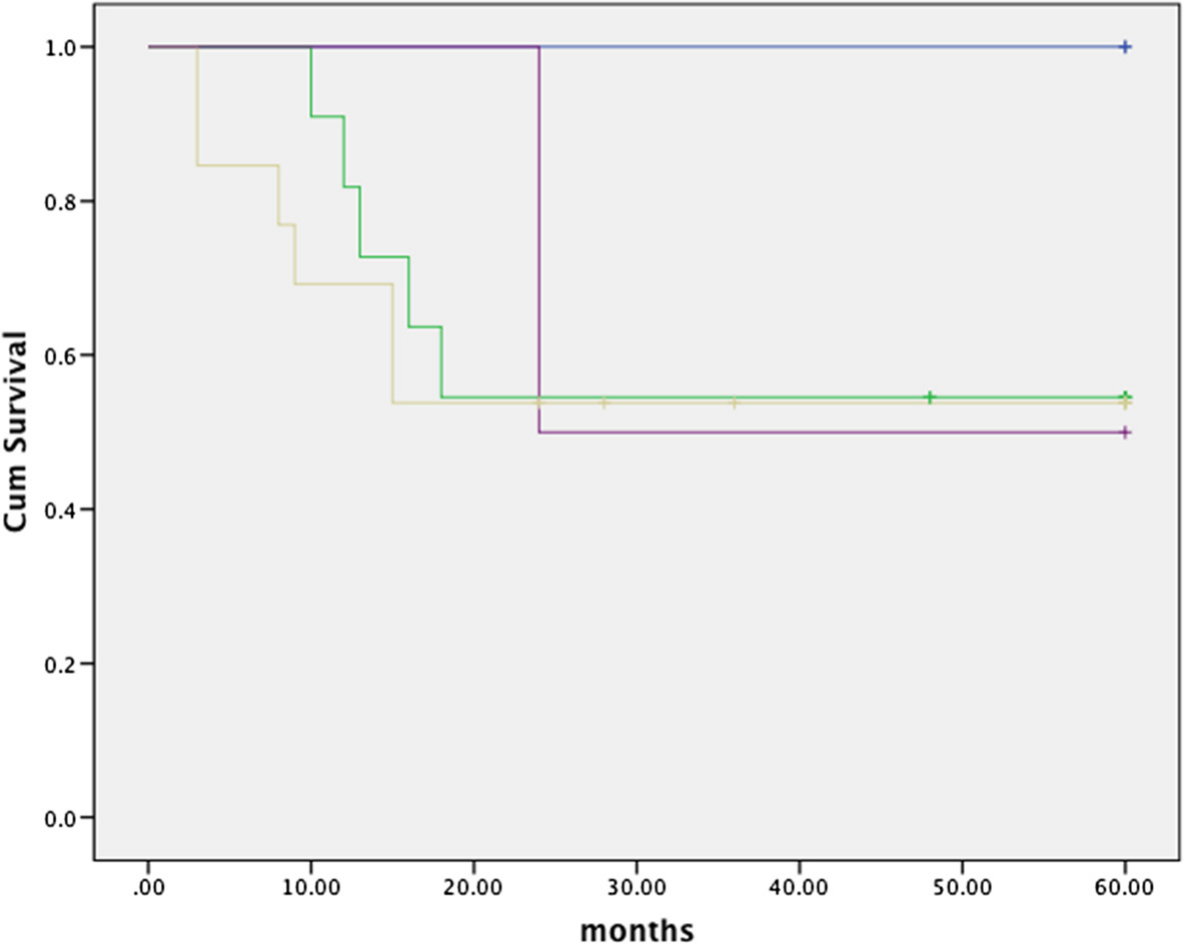
Kaplan Meier curve showing 5-year survival of OPC patients following surgical salvage; p value not significant: blue: stage II, green: III, yellow: IVA, purple: IVB.

Sixty-three patients treated with curative intent experienced treatment failure: 33 (52.4%) were residual tumors, 23 (36.5%) were recurrent tumors, and 7 (11.1%) were second primaries (Figure 3). Of the 28 patients who presented with recurrent tumors or a second primary, the average time to treatment failure was 16.7 (±15.9) months and ranged from 3 to 60. Thirty-five patients with treatment failure were considered inoperable. Patients were considered inoperable if they presented with advanced disease involving extensive tumor spread and specifically if the tumor involved vital structures (i.e. the major vessels) or distant sites. Furthermore, most of these patients were 60 years of age or older and elected to forgo salvage surgery because they felt that the likelihood of a cure did not outweigh the risk of surgical morbidity. Twenty-eight patients (44.4% of patients who experienced treatment failure) went on to have salvage surgery. Twenty patients underwent a neck dissection in addition to local tumor excision, 7 patients underwent local tumor excision alone, and 1 patient had a neck dissection alone. ^c^ Five-year survival for salvage patients stratified by primary disease stage II-IVB was 100%, 54.5%, 53.8%, and 50%; differences in survival were not statistically significant (p > 0.05). Refer to Figure 4 for 5-year survival of salvage patients. Fifteen (53.6%) patients who underwent surgical salvage were cured of their disease. Of those who recurred following surgical salvage: 4 developed distant tumors, 5 had a locoregional recurrence, 3 developed second primaries, and 1 developed a third primary. The average number of months to failure following salvage was 6.7 (±3.3) and ranged from 2 to 11. Overall survival for these patients was 13.1 (±7.1) months and ranged from 3 to 28.

### Free-flap reconstruction

Of the 28 patients that underwent salvage surgery, 15 (53.6%) underwent free-flap reconstruction. All patients that underwent free-flap reconstruction had undergone primary tumor excision with a neck dissection. The radial forearm was the most common donor site; other free flaps included anterolateral thigh, and subscapular system flaps. There were no intra-operative or postoperative complications associated with free-flap reconstruction, and none of the 15 flaps failed postoperatively. Of the patients that did not undergo free-flap reconstruction, 4 underwent primary tumor excision, 8 underwent primary tumor excision and neck dissection, and 1 underwent a neck dissection alone.

### Comparison of free-flap and non-free-flap patients regarding use of post-salvage PEG-tubes and/or tracheostomies

**Figure 5:**
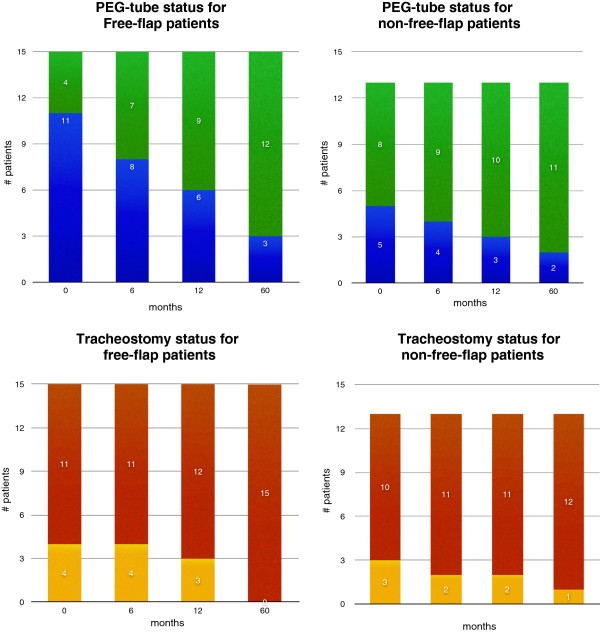
**Top two graphs: number of patients with (blue) and without (green) a PEG-tube at 0, 6, 12, and 60 months post-operative following salvage surgery.** On the left are patients who underwent free-flap reconstruction, and on the right are patients who did not undergo non-free-flap reconstruction. Bottom two graphs: number of patients with (yellow) and without (red) a tracheostomy at 0, 6, 12, and 60 months post-operative following salvage surgery. On the left are patients who underwent free-flap reconstruction, and on the right are patients who did not undergo non-free-flap reconstruction.

At 0, 6, 12 and 60 months following salvage surgery 11 (73.3%), 8 (53.3%), 6 (40%), and 3 (20%) free-flap patients had a PEG-tube; and 5 (38.4%), 4 (30.8%), 3 (23.1%), and 2 (15.4%) non-free-flap patients had a PEG-tube. At 0, 6, 12 and 60 months following salvage surgery 4 (30.1%), 4 (30.1%), 3 (23.1%), and 0 free-flap patients had a tracheostomy; and 3 (23.1%), 2 (15.4%%), 2 (15.4%), and 1 (7.8%) non-free-flap patients had a tracheostomy. Tracheostomy and PEG-tube status of patients with and without a free flap is depicted in Figure 5. Relative risk (RR) with a 95% confidence interval was used to determine whether the need for a PEG-tube or tracheostomy was different between patients who underwent free-flap reconstruction compared to those who did not. Patients who underwent free-flap reconstructions were consistently more likely to require a PEG-tube at 0, 6, 12, and 60 months of clinical follow-up, however, these results did not reach statistical significance (p > 0.05). Similar results were obtained for use of tracheostomy, though the risk was reversed at 60 months of clinical follow-up; RR was not statistically significant (p > 0.05).

## Discussion

The sample of OPC patients included in this study was predominantly composed of males, aged 60 year or older, with a history of smoking and/or drinking. The majority of patients who presented with oropharyngeal primaries were treated with curative intent. Only a small number of patients were considered incurable, and this was due to the advanced nature of their disease. Surgery as first-line treatment was rare and was only performed in one patient. ^d^ This finding aligns with current trends in OPC treatment whereby RT or CCRT is often favored over surgery as first-line approach [1,5]. In this study, CCRT was administered more frequently than RT. This result reflects the fact that the majority of OPC patients presented with regional involvement (N ≥ 2), and chemotherapy is thought to be the best modality for the management of OPC with neck disease. This analysis revealed that the rate of residual disease was higher for patients who underwent CCRT compared to those who underwent RT. Because patients who received CCRT typically presented with more advanced disease than those treated with RT, the higher incidence of residual disease is likely reflective of disease stage rather than treatment modality. In contrast, despite the fact that regional metastasis is associated with a higher risk of recurrence, and patients in the CCRT group generally presented with more advanced neck disease, the rate of recurrence was significantly lower for the CCRT group compared to the RT group. This finding supports the notion that chemotherapy is superior to RT for the management of nodal metastasis. This investigation did not find a significant difference in the length of disease-free survival between the two treatment groups; however, the lack of significant findings may have been attributed to the degree of variability in the time to recurrence within each group.

Of all patients who experienced treatment failure, just over half were amenable to surgery. There are a number of variables that factored into the decision to proceed with salvage surgery. In order for patients to be eligible for salvage surgery, tumors must be minimally invasive and cannot involve vital structures (i.e. major vessels) and/or multiple/distant sites, and patients must present with minimal comorbidity and a moderate to high functional status rating. While the majority of salvage patients were cured of their disease following salvage procedures, the failure rate was relatively high at 39%.

According to Cohen et al., it is still unclear whether CCRT followed by salvage surgery is really more effective than surgery followed by adjuvant CCRT [1]. Proponents of the organ-sparing approach argue that administering CCRT as first-line treatment offers patients a chance of a cure while sparing them from the risk of surgical morbidity. However, if close to half of patients fail following CCRT then many patients still end up requiring surgery. The issue is that by the time patients fail following CCRT only about half are eligible for further treatment, and out of those who undergo surgical salvage, only about half will be cured of their disease.

Since the introduction of free flaps in the late 80’s, postoperative functional rehabilitation has significantly improved [6]. According to Stoker et al. combined-modality treatment of OPC that includes free-flap reconstruction enhances post-salvage outcomes by improving airway management. In this study, the risk of PEG-tubes and tracheostomy was comparable for those who underwent free-flap construction compared to those who did not. Though this finding seems to contradict previous findings demonstrating that free-flap reconstruction is associated with improved functional rehabilitation. Our results may have been a consequence of the small sample size or due to the fact that most of the patients undergoing free-flap reconstruction were subjective to more invasive procedures. It is worth pointing out that despite the fact that the instance of PEG-tube use was higher among free-flap patients, there were more free-flap patients who had their PEG-tube removed over the course of 60 months. Because this study did not include a valid comparator for free-flap patients, the therapeutic advantage of free-flap reconstruction could not be directly quantified; however, it is very possible that those who received a free flap were rid of their PEG-tube and/or tracheostomy sooner than they would have if they had not received a free-flap.

## Conclusion

While the modality of choice for first-line treatment of OPC is CCRT, moderate failure rates suggest that selected patients may be better off with surgery as first-line treatment. While treatment-related morbidity is a concern with surgery, it is important to point out that not all surgeries are associated with a high risk of morbidity. For instance, it may be the case that less invasive procedures such as transoral laser surgery with integrated neck dissection are better tolerated and could therefore be regarded as a reasonable alternative to CCRT. Continued research comparing the efficacy of different therapeutic approaches involving a combination of surgery and CCRT is needed and on the therapeutic potential of free-flap reconstruction in patients undergoing surgical salvage for recurrent or residual OPC primaries is needed.

## Endnotes

^a^Yearly mortality rates specific to oropharyngeal cancer (OPC) are currently unavailable.

^b^Five-year survival for disease stages II through IVB was 71.4%, 64.3%, 57.0%, and 40%. Differences in survival proportions by disease stage were statistically significant (p = 0.05).

^c^There were 8 patients who underwent an salvage neck dissection following CCRT, however, surgical pathology did not come back showing residual disease, therefore even though these patient underwent salvage surgery they were not considered to have treatment failure and were therefore excluded from the following analysis. The vast majority of neck dissections were unilateral modified radical.

^d^This patient presented with significant boney invasion of the mandible and required an immediate manibuloectomy. Surgery was followed by adjuvant CCRt. This patient had a complete response and was cured of their disease.

## Abbreviations

OPC: Oropharyngeal cancer; SHSC: Sunnybrook Health Science Centre; RT: Radiotherapy; CCRT: Concomitant chemoradiotherapy; DOD: Dead of disease; NED: No evidence of disease; AWD: Alive with disease.

## Competing interests

There are no competing interests to declare. This project is uncompensated and none of the authors have commercial, political, personal or ideological interests to disclose; nor are any of the investigators affiliated with an organization that has financial, political or ideological investments in this study.

## Authors’ contributions

LW was involved in study design including developing inclusion criteria, selecting eligible patients, identifying parameters of interest, and designing an appropriate statistical plan. LW was also involved in data collection, analysis, and interpretation. LW drafted the manuscript, carried out necessary revisions, and readied the manuscript for submission. KH made substantial contributions to the design of the study relating to inclusion criteria, identifying parameters of interest, and interpreting research results. KH was also responsible for editing the manuscript, and submitting it for publication. DE fulfilled a similar role, contributing to the design of the study and participated in editing the final manuscript. All authors read and approved the final manuscript.
